# Microfluidic Autologous Serum Eye-Drops Preparation as a Potential Dry Eye Treatment

**DOI:** 10.3390/mi7070113

**Published:** 2016-07-04

**Authors:** Takao Yasui, Jumpei Morikawa, Noritada Kaji, Manabu Tokeshi, Kazuo Tsubota, Yoshinobu Baba

**Affiliations:** 1 Department of Applied Chemistry, Graduate School of Engineering, Nagoya University, Furo-cho, Chikusa-ku, Nagoya 464-8603, Japan; morikawa.jumpei@gmail.com (J.M.); kaji@apchem.nagoya-u.ac.jp (N.K.); 2 ImPACT Research Center for Advanced Nanobiodevices, Nagoya University, Furo-cho, Chikusa-ku, Nagoya 464-8603, Japan; tokeshi@eng.hokudai.ac.jp; 3 Japan Science and Technology Agency (JST), PRESTO, 4-1-8 Honcho, Kawaguchi, Saitama 332-0012, Japan; 4 Division of Applied Chemistry, Faculty of Engineering, Hokkaido University, Sapporo 060-8628, Japan; 5 Department of Ophthalmology, School of Medicine, Keio University, Tokyo 160-8582, Japan; tsubota@z3.keio.jp; 6 Health Research Institute, National Institute of Advanced Industrial Science and Technology (AIST), Takamatsu 761-0395, Japan

**Keywords:** dry eye, autologous serum eye-drops, spiral microchannel

## Abstract

Dry eye is a problem in tearing quality and/or quantity and it afflicts millions of persons worldwide. An autologous serum eye-drop is a good candidate for dry eye treatment; however, the eye-drop preparation procedures take a long time and are relatively troublesome. Here we use spiral microchannels to demonstrate a strategy for the preparation of autologous serum eye-drops, which provide benefits for all dry eye patients; 100% and 90% removal efficiencies are achieved for 10 μm microbeads and whole human blood cells, respectively. Since our strategy allows researchers to integrate other functional microchannels into one device, such a microfluidic device will be able to offer a new one-step preparation system for autologous serum eye-drops.

Dry eye is a problem in tearing quality and/or quantity, mainly due to overusing personal computers, tablets, and smartphones, air-drying, and wearing contact lenses. Nowadays, the number of persons suffering from dry eyes may well be over a hundred million worldwide and it increases daily. Dry eyes may be seen as a lack of tears on the corneal epithelial layer induced by corneal damage, and it is also a symptom of problems such as meibomian gland dysfunction and Sjögren’s syndrome. Since the reasons for dry eyes are not straightforward, commercially available eye-drops are generally insufficient to treat dry eyes completely; they only can lubricate the front surface of the eye.

Autologous serum eye-drops are a good candidate for dry eye treatment since they contain epidermal growth factor (EGF), vitamin A, and so on, which is essential for cell differentiation and division [1,2,3]. Treatment using the autologous serum eye-drops is based on the concept that dry eye worsening is not due to drying out the front surface of the eye, but rather to poorly supplying essential components to the cornea; therefore, the autologous serum eye-drops can treat dry eyes comprehensively, by not only lubricating the front surface of the eye but also promoting cornea regrowth by the EGF [4]. Autologous serum eye-drops have two features. One is that users can reduce the chance of infection because the person’s own blood is utilized, and the other is that the eye-drops can be stored for up to three months at −80 °C. The autologous serum eye-drops are prepared as follows: first, a patient’s blood is collected in a heparin-unmodified blood collection tube; secondly, the collected blood is centrifuged at 3000 rpm for 10 min; thirdly, the supernatant is filtered through a 0.45-μm-pore-size filter; and finally, the filtered serum is diluted to reach a target concentration using saline. However, the preparation is relatively troublesome and takes a long time due to the centrifugation, filtration, and dilution steps.

Here we demonstrated a strategy for the preparation of autologous serum eye-drops using a microfluidic technique. Microfluidics has shown great promise for significantly improving diagnostics, as well as biological and medical research studies [5]. Microfluidics has been variously used for passive blood cells separation approaches [6], such as hydrodynamic separation [7,8,9,10,11,12,13,14,15,16,17], sedimentation-based separation [18,19,20,21], and filtration-based separation [22,23,24,25,26,27,28,29,30,31,32,33]. Considering the desire for high throughput and the need for a further dilution process, we fabricated a spiral microchannel (Figure 1a) to realize inertial migration, one of the hydrodynamic separation techniques [34]. In curving microchannels, particles experience a combination of inertial lift force and Dean drag force; inertial lift force acts to focus microbeads at an equilibrium position between the channel wall and centerline [35,36], and Dean drag force acts to entrain microbeads as two counter-rotating vortices with flow directed toward the outer bend at the midline of the channel and inwards at the channel edges [37,38]. A ratio of these forces (inertial lift, *F_L_*/Dean drag, *F_D_*) would be a key parameter to determining the equilibrium positions of the microbeads [39,40]. An inertial force ratio, *R* = *F_L_*/*F_D_* ≈ *a*^3^ ≈ 1/*H*^3^, where *a* is the particle diameter and *H* is the channel height, is obtained by dividing the dimensional scaling of the inertial lift force with the scaling of the Dean drag force [13,40,41]. This force ratio shows that particles with a larger diameter migrate to inertial equilibrium positions, and particles in a channel of larger height do not migrate to inertial equilibrium positions but remain entrained in the channel vortices. We demonstrated the focusing of 10-μm-diameter microbeads (2.65%, Polyscience, Inc., Warrington, UK) at the equilibrium position close to the inner wall of the spiral microchannel (Figure 1b). Using the spiral microchannel, we performed blood cell removal for the microfluidic autologous serum eye-drops preparation as a potential dry eye treatment.

For the fabrication of microfluidic devices with a spiral microchannel, we used poly(dimethylsiloxane) (PDMS; silpot 184, Dow Corning Toray Co., Ltd., Tokyo, Japan) replication techniques from an SU-8 mold (SU-8 3050, Nippon Kayaku Co., Ltd., Tokyo, Japan). First, photo-curable SU-8 resin was spin-coated on Si substrates (Silicon Technology Co., Ltd., Tokyo, Japan) and pre-baked at 95 °C for 20 min. The thickness of the SU-8 resin was controlled by spinner rotation speed and time. The SU-8 microchannel was patterned by a mask aligner (MJB4, SÜSS MicroTec AG., Munich, Germany) through emulsion photomasks (Topic Co., Ltd., Kawaguchi, Japan). In addition, the patterned SU-8 resin was post-baked at 95 °C for more than 4 min and developed using a SU-8 developer (Nippon Kayaku Co., Ltd.). The developed SU-8 mold was finished by putting it into a vacuum chamber under a trichloro(1H, 1H, 2H, 2H-perfluorooctyl)silane atmosphere for 3 h. PDMS was poured into the silanized SU-8 mold and cured at 80 °C for 2 h. After peeling off the cured PDMS, via holes were made for one inlet and two outlets. The PDMS with the via holes and glass slides were bonded to each other after plasma treatment (SDP-1012, Meiwafosis Co., Ltd., Tokyo, Japan). Removal efficiency (collection efficiency) was calculated by dividing the number of introduced microbeads or blood cells by collected ones. In addition, the number of microbeads or blood cells was calculated using collected sample volume and concentrations, which are estimated from a calibration curve (optical density vs. concentrations).

The spiral microchannels showed 100% removal efficiency for 10-μm-diameter microbeads, which is a model material for blood cells (Figure 2). The features of the spiral microchannels, such as the aspect ratio, the number of microchannel spirals, and flow rates, should be candidate parameters governing removal efficiency. Since maximum channel velocity, which is determined by the cross-sectional area of the microchannel, is known to affect removal efficiency [34,40,41,42], we supposed that the cross-sectional area should be 50,000 μm^2^. By changing the aspect ratio from 0.1 to 1.0 under other fixed conditions, we concluded that the aspect ratio from 0.1 to 0.2 was suitable for 10 μm particle removal; in particular, the 0.1 ratio gave a 99% removal efficiency (1% collection efficiency) at the outer outlet (Figure 2a). This meant that a smaller aspect ratio had higher removal efficiency, which was in good agreement with the behavior predicted by the inertial force ratio: particles in a smaller height channel migrated to inertial equilibrium positions. Next, we considered the effect of the number of microchannel spirals, ranging from 0.5 to 7.5 spirals, on removal efficiency (Figure 2b). Figure 2c showed that the removal efficiency increased with an increase of the number of microchannel spirals, leading to 99% removal efficiency (1% collection efficiency) at one outer outlet in 7.5 spirals. From the above results, we used the spiral microchannel with a 0.1 aspect ratio and 7.5 spirals to examine influence of flow rates on removal efficiency (Figure 2d). As we increased the flow rate from 100 to 5000 μL/min, the removal efficiency drastically improved, and finally we achieved 100% removal efficiency (0% collection efficiency) at the flow rate of 5000 μL/min.

Finally, we introduced whole human blood into the spiral microchannels and achieved 90% removal efficiency of blood cells at the outer outlet (10% collection efficiency) (Figure 3). After sampling and centrifugation of whole human blood, we mixed blood cells and blood plasma to be 50% hematocrit, and then we diluted the blood sample using phosphate buffered saline to reach target hematocrit values. As for the 10 μm microbeads, the removal efficiency of blood cells increased as the flow rate increased; however, we could not attain 100% efficiency due to the disc shape of the red blood cells which had an 8 μm diameter and 2.5 μm thickness (Figure 3a). Considering the inertial force ratio, it made sense that removal efficiency was degraded for the smaller particle diameter. It is well known that the inertial lift force drops with a decrease in the Reynolds number [34,35,36], and as we expected, the viscosity of the blood samples affected removal efficiency, and the removal efficiency at the outer outlet increased to 90% (10% collection efficiency) as the concentration decreased (Figure 3b). Figure 3c shows photographs of collected blood samples at the inner and outer outlets; hemolyzed blood was not observed. We confirmed that hemolyzed blood was not observed at any of the concentrations used (Figure 3b). From these results, we concluded that inertial force in the spiral microchannels at the concentrations used had no hemolyzing property.

To achieve the 100% removal efficiency of blood cells, we can propose two methods: increasing the inertial lift force and decreasing the Dean drag force. Both ways lead to increasing the inertial force ratio. For increasing the inertial lift force, we should increase the Reynolds number by increasing the flow rates [36,43,44]. In this approach, we could apply 10,000 μL/min for a maximum flow rate due to a deformability issue of PDMS. Since Si, glass or polymethyl methacrylate (PMMA) are much harder materials than PDMS, these microchannels can be good candidates for applying more than 10,000 μL/min. Note that we should confirm the hemolysis issue of blood cells when we apply more than 10,000 μL/min. For decreasing the Dean drag force, we should decrease the Dean number by reducing the channel height or increasing the curvature ratio [38,39,40]. In this approach, we used the microchannels with a 0.1 aspect ratio and 7.5 spirals due to a roof collapse issue of PDMS and a size issue of glass slides. Si, glass or polymethyl methacrylate (PMMA) microchannels would also help researchers to avoid the roof collapse issue and reduce the aspect ratio, and a larger size of the glass slides would allow researchers to avoid the size issue and increase the number of microchannel spirals. Note that we should confirm a clogging issue of blood cells when we use lower aspect ratio microchannels.

In summary, we have demonstrated a strategy for the preparation of autologous serum eye-drops based on spiral microchannels, which enables passive blood cell removal. The spiral microchannels achieved complete removal of 10 μm microbeads as a model sample, and 90% removal of whole human blood cells. While the current removal efficiency is not yet enough to make autologous serum eye drops, flow rates with more than 10,000 μL/min (up to a flow rate without hemolysis), which can increase the inertial lift force, and lower aspect ratio microchannels (down to an aspect ratio without clogging) over eight spirals, which can decrease the Dean drag force, have the potential for application in preparation devices for blood cell removal, with the eventual goal of realizing the dry eye treatment. Since the present strategy allows researchers to make a further integration with a separation microchannel for platelets and clotting factors and a dilution microchannel, such microfluidic devices can offer a new path for the development of a one-step preparation system for autologous serum eye-drops.

## Figures and Tables

**Figure 1:**
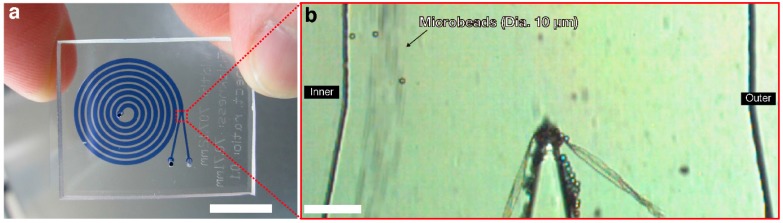
A spiral microfluidic device. (**a**) Photograph of a spiral microfluidic device; scale bar, 10 mm. Microchannels are highlighted by Trypan blue dye solution. Channel width and height are 707 and 70.7 μm, respectively. Distance between two adjacent microchannels is 303 μm; (**b**) A magnified micrograph of part of a spiral microchannel, enclosed by the red dotted box in Figure 1a; scale bar, 100 μm. Ten-fold diluted microbeads (10 μm diameter) in phosphate buffered saline were focused at an equilibrium position close to the inner wall of the microchannel.

**Figure 2:**
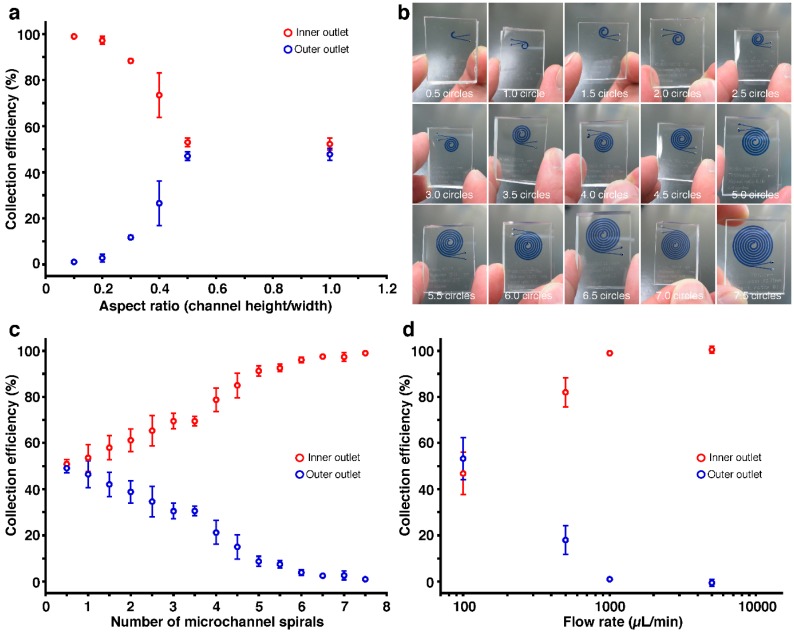
Collection efficiency of 10 μm particles. Cross-sectional area was 50,000 μm^2^. Ten-fold diluted microbeads (10 μm diameter) in phosphate buffered saline were used. Error bars are the standard deviation for a series of measurements (*N* = 3). (**a**) Collection efficiency vs. aspect ratio of spiral microchannels. The aspect ratio is the ratio of channel height to width. The number of microchannel spirals was 7.5, and flow rate was 1000 μL/min; (**b**) Photographs of fabricated spiral microchannels with 0.5 to 7.5 circles. One circle is one spiral. The microchannels are highlighted by Trypan blue dye solution; (**c**) Collection efficiency vs. number of microchannel spirals. The aspect ratio of the microchannels was 0.1, and flow rate was 1000 μL/min; (**d**) Collection efficiency vs. flow rate. The aspect ratio of the microchannels was 0.1, and the number of microchannel spirals was 7.5.

**Figure 3:**
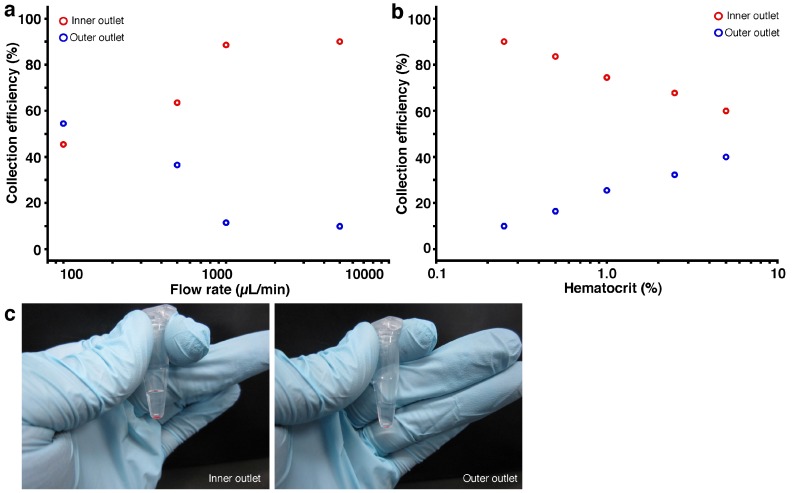
Collection efficiency of whole human blood cells. Cross-sectional area was 50,000 μm^2^, the aspect ratio was 0.1, and the number of microchannel spirals was 7.5. (**a**) Collection efficiency vs. flow rate. Initial hematocrit of blood samples was 0.25%; (**b**) Collection efficiency vs. whole blood concentration. Flow rate was 5000 μm/min; (**c**) Photographs of collected samples from inner and outer outlets after centrifugation. Flow rate was 5000 μm/min, and initial hematocrit of blood samples was 0.25%. Hemolyzed blood was not observed.
